# Internal state affects local neuron function in an early sensory processing center to shape olfactory behavior in *Drosophila* larvae

**DOI:** 10.1038/s41598-022-20147-1

**Published:** 2022-09-21

**Authors:** Seth R. Odell, David Clark, Nicholas Zito, Roshni Jain, Hui Gong, Kendall Warnock, Ricardo Carrion-Lopez, Coral Maixner, Lucia Prieto-Godino, Dennis Mathew

**Affiliations:** 1 Integrative Neuroscience Program, University of Nevada, 1664 N. Virginia St., MS: 0314, Reno, NV 89557 USA; 2 Molecular Biosciences Program, University of Nevada, Reno, NV 89557 USA; 3 The Francis Crick Institute, London, NW1 1AT UK; 4 Department of Biology, University of Nevada, Reno, NV 89557 USA; 5 NSF-REU (BioSoRo) Program, University of Nevada, Reno, NV 89557 USA

**Keywords:** Sensory processing, Neural circuits

## Abstract

Crawling insects, when starved, tend to have fewer head wavings and travel in straighter tracks in search of food. We used the *Drosophila melanogaster* larva to investigate whether this flexibility in the insect’s navigation strategy arises during early olfactory processing and, if so, how. We demonstrate a critical role for Keystone-LN, an inhibitory local neuron in the antennal lobe, in implementing head-sweep behavior. Keystone-LN responds to odor stimuli, and its inhibitory output is required for a larva to successfully navigate attractive and aversive odor gradients. We show that insulin signaling in Keystone-LN likely mediates the starvation-dependent changes in head-sweep magnitude, shaping the larva’s odor-guided movement. Our findings demonstrate how flexibility in an insect’s navigation strategy can arise from context-dependent modulation of inhibitory neurons in an early sensory processing center. They raise new questions about modulating a circuit’s inhibitory output to implement changes in a goal-directed movement.

## Introduction

Insects navigate complex odor environments containing the smells of predators and food. During navigation, they evaluate the direction of various odor sources before making navigation decisions. For instance, the *Drosophila* larva uses head sweeps to assess the surrounding odor gradient before steering to the left or right^1^. Furthermore, the insect’s satiety state influences such adaptive decisions. For example, during the crawling stages of many insect species, starved subjects tend to have fewer head wavings and straighter tracks than their satiated counterparts^2–5^.

Several adaptive behaviors in insects originate in the activities of neurons in the antennal lobe, the first olfactory processing center^6^. This processing center receives axonal projections from odor-sensing first-order olfactory sensory neurons (OSNs). Here, OSNs connect with second-order projection neurons and local neurons^7^. The insect’s satiety state modulates the functions of neurons in this first processing center^6,8–10^. Recent studies have revealed that hunger affects the functions of first-order sensory and second-order projection neurons in the *Drosophila* antennal lobe^6,11–14^. However, it is unclear whether hunger affects the functions of local neurons in the antennal lobe (AL), a knowledge gap we explore in the present study.

Here, we focus on the hunger-dependent modulation of Keystone-LN, a local neuron in the *Drosophila* larva’s AL. The *Drosophila* larva is ideal for exploring these mechanisms because of new research tools and the long history of research on this organism^1,15–22^. Furthermore, unlike other olfactory systems, the *Drosophila* larval AL has fewer neurons^7,22–26^. Preliminary studies from our lab indicated that Keystone-LN activity influences head wavings in the larva. Recent EM reconstruction and mapping studies determined that Keystone-LN occupies a central position in the *Drosophila* antennal lobe circuits. It connects with first-order OSNs, second-order projection neurons, and neurons in the sub-esophageal zone (SEZ)^7^. Previous studies have shown descending neurons in the SEZ contact premotor neurons responsible for head movements^27–29^. We hypothesized that Keystone-LN responds to odor stimulation and state-dependent modulation to coordinate head-sweep behavior, an adaptive behavior in the *Drosophila* larva.

We aimed to understand how an animal’s satiety state influences navigational flexibility. First, we confirmed that starved *Drosophila* larvae have fewer head sweeps than their satiated counterparts. We discovered that Keystone-LN, an inhibitory local neuron in the larval antennal lobe, triggers head-sweep behavior. Next, we explore Keystone-LN’s responses to odor and its role in larval olfactory behavior. Finally, we show that the larva’s satiety state affects the magnitude of Keystone-LN-induced head sweeps, likely mediated by altering insulin signaling levels. In summary, we show that a larva’s satiety state affects an inhibitory local neuron’s role in head-sweep behavior, shaping the larva’s odor-guided movement. These results provide new insights into how internal contexts influence an olfactory circuit to control adaptive decisions shaping odor-guided navigation.

## Results

### A larva’s satiety state affects its head-sweep behavior

**Figure 1:**
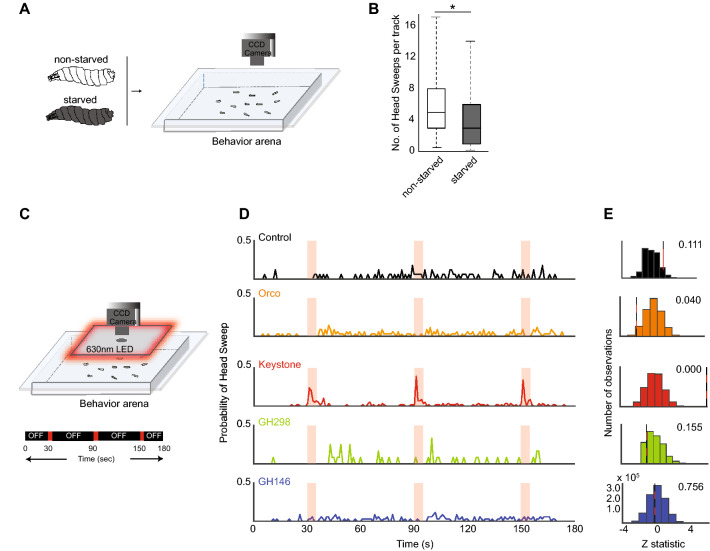
Keystone-LN activity triggers head-sweep behavior in the *Drosophila* larva. (**A**) Tracking assay. Starved and non-starved individuals of wild-type third-instar *Drosophila* larvae are imaged in the tracking assay. (**B**) The average number of head sweeps for each larval track is plotted on the -axis. Mann–Whitney *U*, *p < 0.05, n = 37 for non-starved condition, n = 47 for starved condition. (**C**) Optogenetics assay. Larval neurons expressing *CsChrimson* are activated by shining red-light on the behavior arena. Larval movements are recorded. The experimental paradigm is shown below the arena. Red-light stimulus is turned ON for 5 s at 30 s, 90 s, and 150 s. (**D**) The probability of head sweeps is plotted on y-axes for control larvae expressing no *CsChrimson* in any neurons (black) and for larvae expressing *CsChrimson* in Keystone-LN (red), GH146 subset of LNs (blue), GH298 subset of LNs (green), and all Orco-positive OSNs (orange). (**E**) Histograms of the calculated Z-statistics between a random 15 s and the remaining 165 s, repeated 1,000,000 times. Red and black lines represent the true Z-statistic between light ON and light OFF. Next to each red and black line is the fraction of replicates with a value greater than the true value.

We surveyed the insect literature to identify conserved patterns in olfactory behavior during starvation. We noted that starved subjects of several crawling insects tend to have fewer head wavings during navigation^2–5^. We wanted to confirm this observation in a model organism such as the *Drosophila* larva with the ultimate goal of elucidating the molecular and cellular basis of such adaptive behavior patterns. We used a tracking assay to analyze larval navigation (Fig. 1A)^1,24^. Specifically, we assessed the average number of head sweeps per larval track (Fig. 1B). Starved larvae had fewer head sweeps during their runs (3.6, 1–6 head sweeps; mean, IQR, n = 47) than non-starved larvae (5.7, 3–8 head sweeps; mean, IQR, n = 37) (Mann Whitney *U*, p < 0.05) (Fig. 1B).

### Keystone-LN activity triggers head-sweep behavior in the *Drosophila* larva

Do any neurons in the larval antennal lobe trigger head-sweep behavior? To address this question, we took advantage of an optogenetic technique developed in our laboratory (Fig. 1C)^21^. This technique allows us to use red light to activate target neuron(s) expressing *CsChrimson* in intact larvae and simultaneously track larval behavior. We calculated the probability of initiating larval head sweeps during multiple 5 s red-light stimuli in control larvae (no *CsChrimson* expression in any larval neuron) and larvae expressing *CsChrimson* in olfactory sensory neurons (*Orco-Gal4*, orange), different sets of local neurons (*Keystone-Gal4* red*, GH298-Gal4*, blue), and projection neurons (*GH146-Gal4*, green) (Fig. 1D,E). *Orco-Gal4* drives expression in all 21 larval OSNs^25,30^. *Keystone-Gal4* drives expression in a single pair of local neurons, Keyston-LN^7^. GH298*-Gal4* drives expression in a cluster of 30–32 (non-Keystone) GABAergic LNs in the larval antennal lobe^31^. GH146-Gal4 drives expression in about two-thirds of all PNs in the larval antennal lobe^32^.

We noted that 5 s stimulation of Keystone-LN consistently increased the probability of initiating head sweeps. However, similar stimulation of other antennal lobe neurons tested here did not increase the probability of initiating head sweeps (Fig. 1D). A randomization test was carried out to determine whether this effect of Keystone-LN activation on the initiation of head turns was significant. We calculated Z-statistic scores between “lights ON” and “lights OFF” frames for control and the four test strains. The Z-statistic value for Keystone-LN (6.04) test strain was the only one significantly greater than 99% of random cases (p < 0.001, Randomization test). The Z-statistic values for the other test strains: GH146 (0.276, p = 0.945), GH298 (− 1.07, p = 0.258), and Orco (− 2.02, p = 0.100) were not significantly greater than random (Fig. 1E). These results suggest that activation of Keystone-LN, and not any other olfactory neurons tested, initiates head sweeps in larvae.

**Figure 2:**
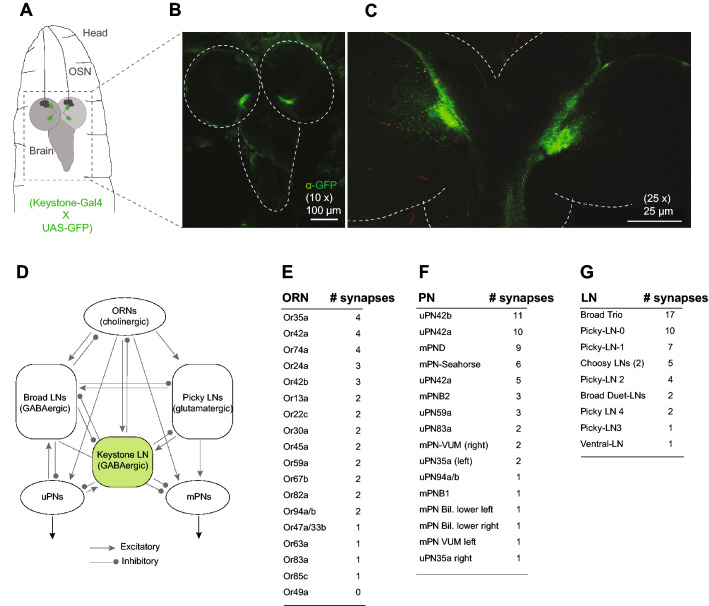
Keystone-LN is centrally positioned in the *Drosophila* larval antennal lobe. (**A**) Cartoon showing the anterior end of a third-instar *Drosophila* larva. Confocal images of the larval brain were taken with a 10 × objective (scale bar is 100 µm) (**B**) and a 25 × objective (scale bar is 25 µm) (**C**). Anti-eGFP antibody stains a single pair of neurons in the two anterior brain lobes (**B**,**C**) and none in the ventral nerve cord (**B**). (**D**) Peripheral olfactory circuit of the *Drosophila* larva. Excitatory and inhibitory connections between Keystone-LN, OSNs, Broad and Picky LNs, uPNs, and mPNs are shown. Adapted from^7^. (**E**–**G**) The number of synapses between Keystone-LN and different classes of neurons is tabulated for (**E**) OSNs, (**F**) PNs, and (**G**) LNs.

To validate the *Keystone-Gal4* line used here, we crossed the *Keystone-Gal4* line with a *UAS-eGFP* line. Next, we dissected third-instar larval progeny and stained the preparations with an anti-GFP antibody. Confocal images confirmed that a single pair of neurons are labeled in the anterior lobes of the third-instar larval brain and none in the ventral nerve cord (Fig. 2A–C). Thus, the expression driven by the *Keystone-Gal4* line used in our study is restricted to a single pair of neurons in the anterior lobes of the third-instar larval brain. These results complement the EM-reconstructions of Keystone-LN using the *Keystone-Gal4* line^7^.

To understand Keystone-LN projections in the larval olfactory circuit, we redrew a Keystone-LN connectivity map and detailed relevant synapse counts based on EM reconstruction studies conducted by^7^ (Fig. 2D). Keystone-LN receives excitatory cholinergic input from almost the entire repertoire of larval OSNs. It makes inhibitory connections with a select set of first-order OSNs and second-order projection neurons (PN) (8 uni-glomerular PNs and 8 multi-glomerular PNs) (Fig. 2E,F)^7,33,34^. Interestingly, Keystone-LN has high connectivity with OSN expressing Or42a, a receptor sensitive to attractive odorants such as 4-hexen-3-one and the corresponding uniglomerular PN: uPN42a. In contrast, Keystone-LN has no direct synaptic connections to OSN expressing Or49a, a receptor sensitive to the aversive odorant menthol (Fig. 2E). Keystone-LN also connects with a subset of local neurons (LNs) (Broad LNs, Choosy LNs, and Picky LNs) (Fig. 2G)^7^. This suggests that Keystone-LN occupies a central position in the *Drosophila* larval AL circuit.

### Odor-evoked calcium responses in Keystone-LN

**Figure 3:**
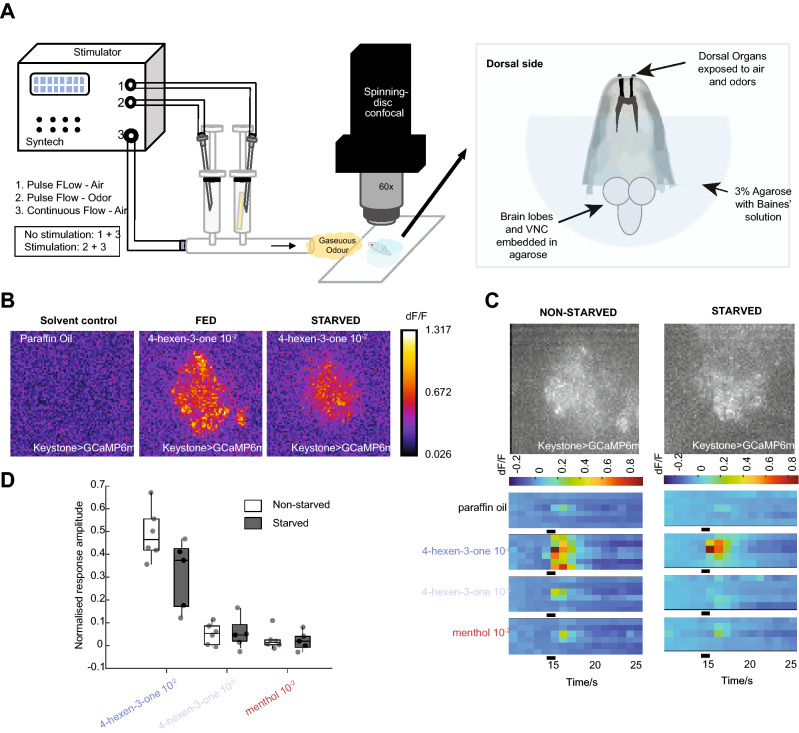
Odor responses of Keystone-LN in fed and starved larvae. (**A**). Schematic of in-vivo calcium imaging set-up. The odor stimulator consists of 3 air outlets, one for the carrier gas flow and two for the pulse flow. Under no stimulus conditions, airflow from outlets 1 and 3 are constant. When a stimulus is triggered, the airflow from outlet 1 is switched to outlet 2, which puffs air through an odor-containing syringe for 1 s, while carrier flow remains constant. Odor responses are recorded from the neurites of Keystones in the AL (Keystone-gal4; UAS-GCaMP6m) using a spinning disk confocal microscope. (**B**) Representative odor-evoked calcium responses in the neurites of Keystone-LNs to the indicated odors diluted in paraffin oil. (**C**) The top panels show maximum projection images of baseline GCaMP fluorescence in Keystone-LN neurites in the antennal lobe. The bottom heatmaps represent the responses of Keystone-LN neurites to the indicated odors under fed and starved conditions. Each row corresponds to responses from an individual larva. The black bar indicates when the 1-s odor stimulus was applied. (**D**) Box plots showing solvent corrected response amplitudes with min–max normalization (see “Methods”) comparing responses to different odors in fed and starved states. Mann–Whitney *U*-test was used to test differences between fed and starved conditions. No statistically significant differences were found for any of the odors tested.

Does odor stimulation of a larva elicit physiological activity in Keystone-LN, and does the animal’s satiety state influence the odor response? We recorded calcium dynamics in Keystone-LNs by imaging GCaMP6m, a genetically-encoded calcium indicator (Fig. 3A). We quantified odor-evoked activity in Keystone-LN neurites in the larval antennal lobe. We tested a high (10^–2^ vol:vol) and a low (10^–5^ vol:vol) concentration of the attractive odor, 4-hexen-3-one, and a high (10^–2^ vol:vol) concentration of the aversive odor, menthol (Fig. 3B–D). Keystone-LN responds strongly to a high concentration of 4-hexen-3-one and weakly to a low concentration of 4-hexen-3-one and to menthol (Fig. 3C,D). There was no statistically significant change in the amplitude of the odor-evoked Keystone-LN response after food deprivation. However, responses to the high concentration of 4-hexen-3-one were slightly decreased in the starved condition (Fig. 3D). These results suggest that specific odors and odor concentrations elicit robust activity in Keystone-LNs.

### Keystone-LN-induced behavior in the presence of odors

**Figure 4:**
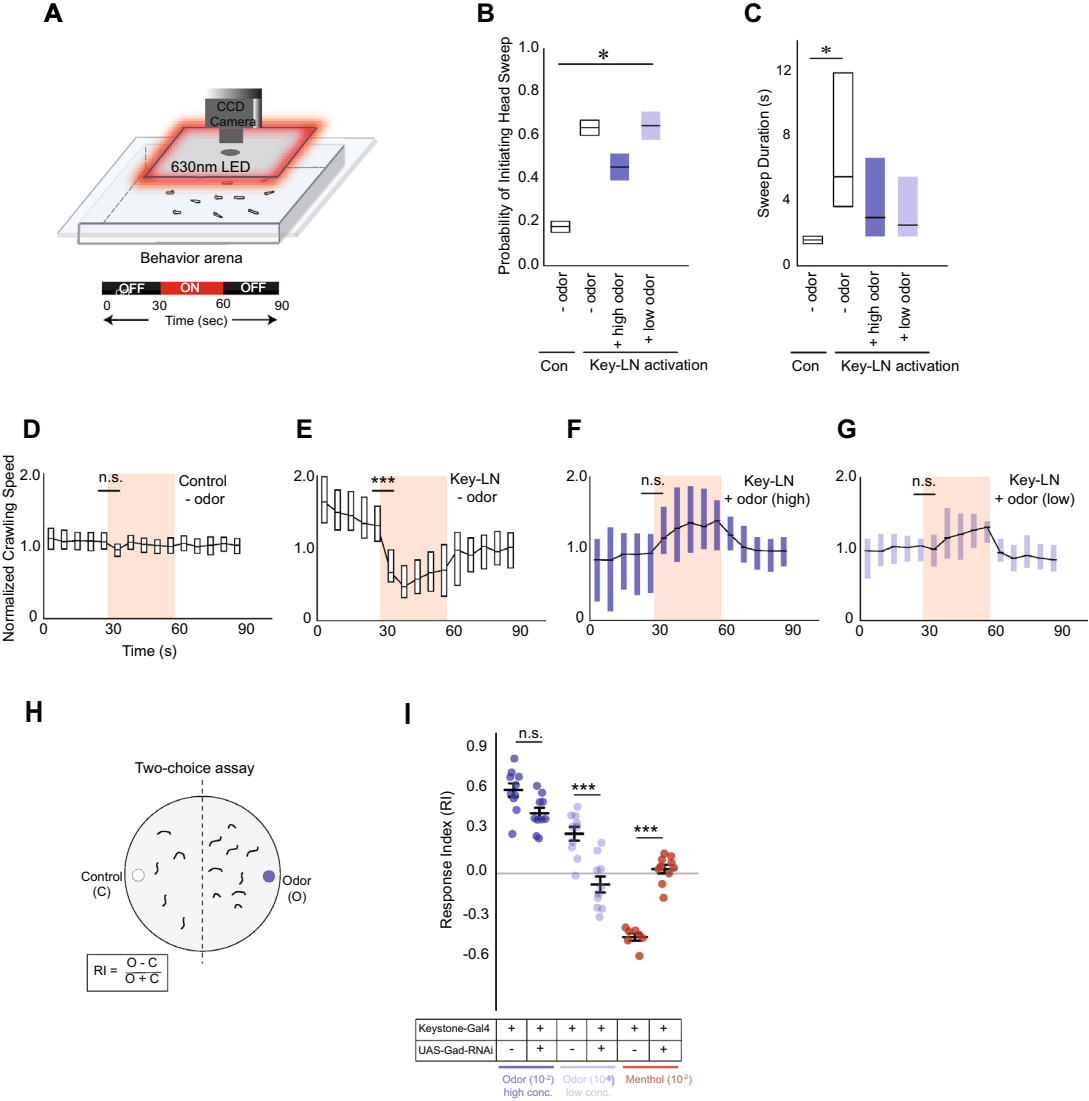
Keystone-LN-induced behavior in the presence of odor. (**A**) Transgenic larvae expressing *CsChrimson* in Keystone-LN are subjected to the optogenetics assay in the absence or presence of an odor (4-hexen-3-one). (**B**) The probability of head sweeps is plotted on the y-axis for control larvae expressing no *CsChrimson* in any neurons and for larvae expressing *CsChrimson* in Keystone-LN both in the absence or presence of a high (10^–2^ vol:vol, dark blue) and a low (10^–6^ vol:vol, light blue) concentrations of 4-hexen-3-one. Distributions are generated by bootstrap analysis. *p = 2e−16, n = 20,000 for each group. (**C**) The duration of each head sweep is plotted on the y-axis. *p = 0.0001, No odor, − activation, n = 8, No odor, + activation n = 51, High odor, + activation n = 16, Low odor, + activation n = 20. (**D**–**G**) The normalized crawling speed of the larvae over the course of the experiment (90 s) is plotted on the y-axis. Control larvae in the absence of odor (**D**) and larvae expressing *CsChrimson* in Keystone-LN in the absence of odor (**E**), in the presence of high odor concentration (**F**), and in the presence of low odor concentration (**G**) are shown. ***p < 0.001, n = 32. (**H**) 2-choice behavior assay. Attraction to test odor is measured as a response index (RI) based on the number of larvae in each half of the plate. (**I**) RI values (mean, SEM) of control and test larvae expressing *Gad-RNAi* in Keystone-LN are plotted on the y-axis. Responses to a high concentration (10^–2^ vol:vol, dark blue dots) and a low concentration (10^–6^ vol:vol, light blue dots) of an attractive odor (4-Hexen-3-one) and to a high concentration (10^–2^ vol:vol, red dots) of an aversive odor (menthol) are shown. Factorial ANOVA, Tukey posthoc test, ***p < 0.001, n = 10 (parent control); n = 10 (test line).

Since 4-hexen-3-one elicits activity in Keystone-LN, does its presence affect the probability of the Keystone-LN-dependent head-sweep behavior? We tested control larvae (no *CsChrimson* expression in any larval neuron) and larvae expressing *CsChrimson* in Keystone-LN in the presence or absence of odor using the optogenetic technique (Fig. 4A). We tested a high (10^–2^ vol:vol, dark blue) and a low (10^–6^ vol:vol, light blue) concentration of 4-hexen-3-one. The probability of head sweeps increased upon light stimulation regardless of the presence or absence of 4-hexen-3-one (Fig. 4B) (Bootstrap analysis followed by one-way ANOVA, p = 2.00e−16, each group n = 20,000). However, the overall duration of a head sweep was significantly higher in the absence of odor than in the presence of a high or low concentration of odor (Fig. 4C) (Kruskal–Wallis, p = 0.0001, each group n-32). This increase in the duration of head sweeps was reflected in the overall crawling speed of the larva. In the absence of odor, Keystone-LN stimulation decreased the larva’s crawling speed (Fig. 4E). In the presence of high or low odor concentrations, Keystone-LN activation did not slow down the crawling speed of the larva (Fig. 4F,G). These results suggest that Keystone-LN-induced head sweep intensity and resulting crawling behavior are sensitive to the odor environment.

Is Keystone-LN activity required for larval attraction and aversion to odors? To address this question, we reduced the GABAergic function of Keystone-LN (by driving a *Gad1-RNAi construct* to reduce GABA biosynthesis^35^). We used a simple 2-choice assay to test the attractive behavior of test and control animals toward high (10^–2^ vol:vol) or low concentrations (10^–6^ vol:vol) of 4-hexen-3-one (Fig. 4H)^36,37^. Lowering Keystone-LN’s inhibitory function did not affect the larva’s response to the high concentration of the attractive odor (Tukey posthoc test, p = 0.0962, each group n = 10) (Fig. 4I; dark blue data points). However, it did impair the ability of test larvae to successfully navigate toward a low concentration of the odor (Tukey posthoc test, p = 1.44e^−4^, each group n = 10) (Fig. 4I; light blue data points). We also tested the degree of larval aversion to menthol (10^–2^ vol:vol), an aversive odor for *Drosophila* larvae^38,39^. Since menthol is a ligand for OSN::49a, which makes no direct connections to Keystone-LN^7^, and since menthol stimulation elicited a very low physiological response in Keystone-LN (Fig. 3C,D), we did not expect to observe any differences in behavioral response toward this odor. However, decreasing Keystone-LN’s inhibitory activity significantly reduced larval aversion to menthol (Tukey posthoc test, p = 1.41e^−4^ each group n = 10) (Fig. 4I). These results suggest that Keystone-LN’s inhibitory function directly or indirectly influences larval behavior toward odors.

### Satiety state affects Keystone-LN function, likely via insulin signaling

**Figure 5:**
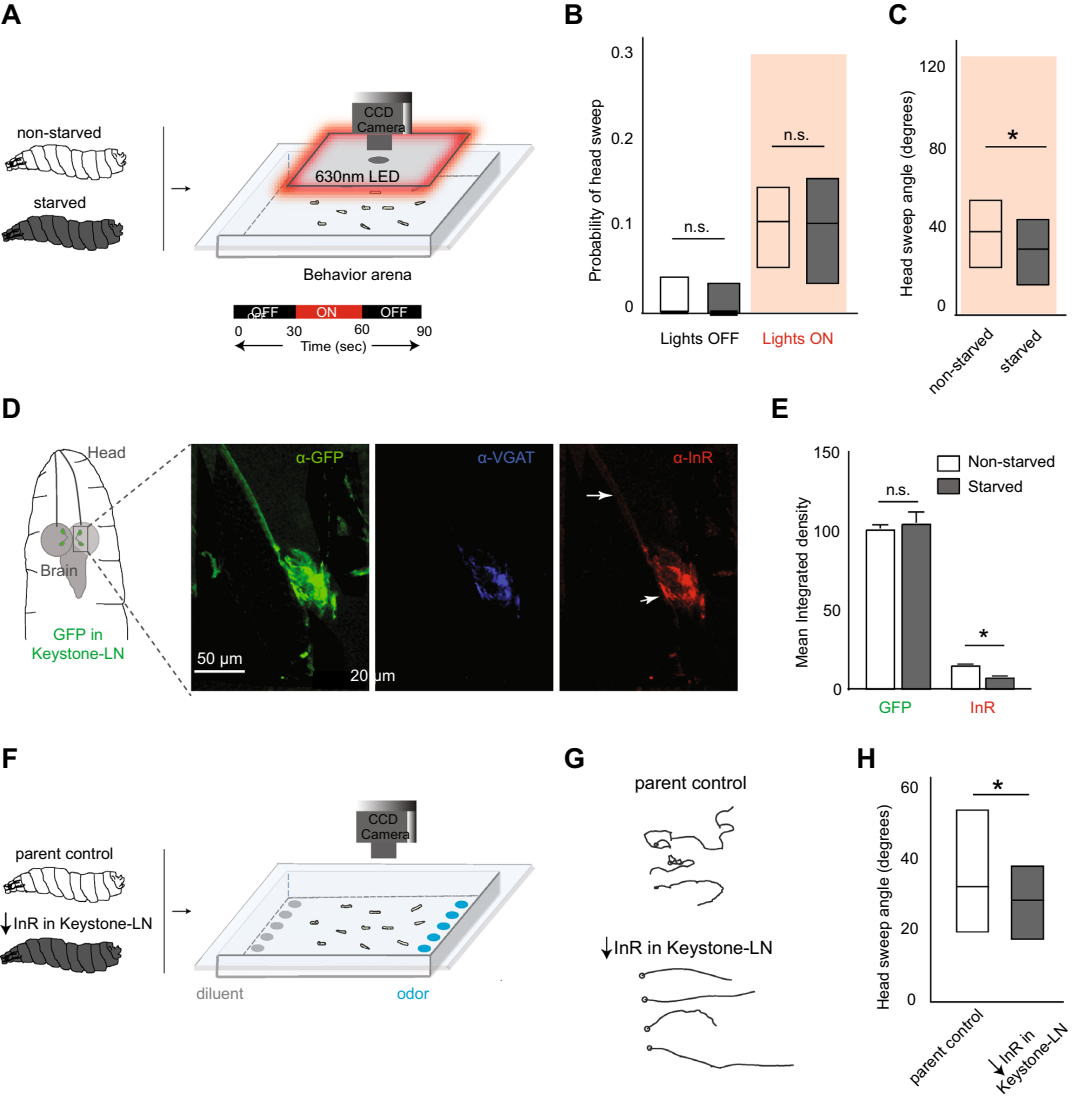
Satiety state and insulin signaling affect head-sweep behavior. (**A**) Starved and non-starved transgenic larvae expressing *CsChrimson* in Keystone-LN are subjected to the optogenetics assay. Larval movements are recorded. (**B**) The probability of head sweeps for non-starved (white) and starved larvae (grey) under lights OFF (left) and lights ON (right) are plotted on the y-axis. Robust non-parametric ANOVA (R-fit package), n = 76 for Lights OFF, n = 15 for Lights ON. (**C**) Magnitudes of head sweep for fed (green) and starved larvae (blue) are plotted on the y-axis. Robust non-parametric ANOVA (R-fit package), *p = 0.0172, n = 72 for fed samples, n = 49 for starved samples. (**D**) Figure depicting the front end of a third-instar *Drosophila* larva. Keystone-LN is highlighted in green. The rectangular inset marks the region of interest during confocal imaging. α-GFP antibody pinpoints Keystone-LN. α-VGAT antibody labels vesicular GABA transporter. α-InR antibody labels insulin receptors. (**E**) GFP and InR protein levels were quantified in Keystone-LNs (mean ± SEM). Student’s *t* test (two-tailed), p < 0.05. (**F**) Transgenic larvae expressing *UAS-InR-RNAi* in Keystone-LN and parent controls are subjected to an odor gradient (4-Hexen-3-one, 10^–2^ vol:vol). Larval movements are recorded. (**G**) Example tracks of control larvae and larvae expressing low InR in Keystone-LNs. (**H**) Magnitudes of head sweep (median, IQR) for control larvae (white, n = 176) and larvae expressing low InR in Keystone-LNs (grey, n = 92) are plotted on the y-axis. Robust non-parametric ANOVA (R-fit package), *p = 0.007.

Starved larvae have fewer head sweeps than non-starved larvae (Fig. 1B). Does the larva’s satiety state directly affect Keystone-LN’s ability to influence head-sweep behavior? We tested larvae that were either starved (two hours, dH_2_0) or fed (two hours, 15% sucrose solution) in the optogenetics assay (Fig. 5A). There was no difference in the probability of head sweeps between fed (white bars) and starved larvae (grey bars). This observation was consistent regardless of whether Keystone-LN was unstimulated (Lights OFF) or stimulated (Lights ON) (Robust non-parametric ANOVA, p > 0.05) (Fig. 5B). However, fed larvae had a significantly higher (> 10°) magnitude of head sweeps than starved larvae (robust non-parametric ANOVA, p = 0.0172, fed: n = 72, starved: n = 49) (Fig. 5C). These results suggest that the animal’s satiety state affects the magnitude of head sweeps but not its probability. Smaller head sweeps could result in starved larvae traveling in straighter tracks. This could allow them to move farther to search for food in less time.

Information about satiety states is typically conveyed via altering insulin levels^8,9,11,13^. Therefore, we tested whether Keystone-LNs express receptors for insulin. We performed immunocytochemistry in a larval line expressing *GFP* in Keystone-LNs. We stained the prep with an α-InR antibody to determine insulin receptor (InR) expression (red channel) (Fig. 5D). We costained the prep with an α-GFP antibody to pinpoint Keystone-LNs (green channel), and α-VGAT antibody to stain vesicular GABA transporter (blue channel) as a further confirmation that we are imaging GABAergic Keystone-LNs (Fig. 5D). These experiments revealed that Keystone-LN expresses InRs in its projections and terminals (white arrows, Fig. 5D). Furthermore, the InR protein expression was sensitive to the larva’s satiety state. Starved animals showed lower InR protein expression in Keystone-LN than non-starved animals (two-tailed Student’s *t* test, p < 0.05) (Fig. 5E). These results suggest that information about satiety states may be conveyed to Keystone-LN via altering insulin or insulin receptor levels or both.

Finally, we asked whether decreasing insulin signaling in Keystone-LN (mimicking starved state) reduces head-sweep magnitudes. And does it influence the shape of larval trajectories? Insulin receptor (InR) expression in Keystone-LNs was reduced by driving an RNAi construct targeting the InR gene using a Keystone-Gal4 driver. This InR-RNAi construct was previously validated and shown to reduce the expression of InR in neurons^12^. First, we performed immunocytochemistry with larval preps expressing both *GFP* and *InR-RNAi* in Keystone-LNs. We stained the preps with an α-BrP (Bruchpilot) antibody to determine the structural integrity of active zones^40^ (Suppl. Fig. 1A). We found that reducing InR expression in Keystone-LNs reduced the number of BrP puncta at Keystone-LN terminals (two-tailed Student *t* test, p = 0.0034, n = 8) (Suppl. Fig. 1A,B). Next, we monitored larval movement using the tracking assay^1,24^ in the presence of an attractive odor (4-hexen-3-one, 10^–2^ vol:vol) (Fig. 5F). Larvae expressing low InR in Keystone-LNs (mimicking starved state, n = 92) had smaller head sweep magnitudes compared to controls (n = 176) (robust non-parametric ANOVA, p = 0.007) (Fig. 5H). They also had straighter tracks toward odors (Fig. 5G). We confirmed this using a metric, ‘curve rating,’ a ratio between the total length of a larva’s track and the actual distance traversed between start and endpoints. Thus, a curve rating of 1 indicates a perfectly linear path. In contrast, higher values indicate a more meandering path toward the endpoint. Larvae expressing low InR in Keystone-LNs had a curve rating close to 1, significantly lower than control animals (Kruskal–Wallis, p = 0.0021, control: n = 19, InR-RNAi: n = 13) (data not shown). These results suggest that insulin signaling likely mediates the satiety-dependent changes in the magnitude of head sweeps to influence the efficacy of odor-guided navigation in larvae.

## Discussion

Local neurons in the insect antennal lobe and the mammalian olfactory bulb play vital roles in early olfactory processing, influencing behavioral outcomes^6^. Here, we demonstrate a critical role for Keystone-LN, a *Drosophila* larval local neuron (Fig. 2), in implementing head-sweep behavior, shaping the larva’s odor-guided navigation (Figs. 1, 4). Keystone-LN responds to odor stimulation (Fig. 3), and Keystone-LN-induced head sweep intensity and resulting crawling behavior are sensitive to the presence or absence of odor (Fig. 4B–G). The larva’s satiety state affects the magnitude of Keystone-LN-induced head sweeps but not their probability (Fig. 5B,C). Keystone-LN expresses insulin receptors (Fig. 5D,E). Insulin signaling levels influence Keystone-LN’s function to calibrate head-sweep behavior and shape larval trajectories (Fig. 5F–H). Head sweeping is an adaptive behavior that allows a larva to evaluate its surrounding odor environment before deciding to steer to the left or right^1^. This study provides new insights into how internal contexts influence olfactory neurons controlling adaptive decisions to shape odor-guided movement. When faced with starvation, it may be adaptive for crawling insects to suppress their head-sweep behavior to travel greater distances in search of food^2–5^.

In general, when the direction of an odor source is clear, larvae tend to have longer, uninterrupted runs toward the odor source. However, when the odor cues are insufficient or less clear, larvae tend to have shorter runs punctuated by stops and head sweeps. Once a *Drosophila* larva stops during chemotaxis, it implements, on average, two high-amplitude head casts before initiating a run^1,41^. Flying insects such as adult *Drosophila* and moths trigger casting behaviors (zig-zag turns perpendicular to odor plume) when unsure of the direction of odor source^42–44^. Vertebrates also sense changes in odor concentration, from sniff to sniff, to guide them to an odor source. In fact, sniffs affect the perception of odor concentration and odor identity^45^. However, we know little about the neural mechanisms that implement situation-based action selections in insects and vertebrates. Thus, our data implicating a local neuron in head-sweep behavior (Figs. 1, 4, and 5) opens up new opportunities to study the neural mechanisms underlying action selections. Furthermore, the *Drosophila* larva holds promise as an excellent experimental system to study these neural mechanisms because available new research tools allow us to access and manipulate its numerically simple and well-defined antennal lobe circuit and simultaneously measure its behavioral output at a high resolution.

Actively orienting toward an odor source necessitates tight coordination between the animal’s sensory input and motor output. Keystone-LN may play a role in this coordination, especially given its connectivity to first-order OSNs, second-order projection neurons, and neurons in the sub-esophageal zone (SEZ)^7^. Previous studies have shown that optogenetic activation of two different descending neurons in the SEZ lead to reorientation behaviors^28,29^. These descending neurons, PDM-DN and SEZ-DN2, contact premotor neurons responsible for head movements^27–29^. Based on morphology and innervation patterns, the Keystone-LN resembles the LN-SEZ local neuron in adult *Drosophila*^46^. Thus, further studies of Keystone-LN function and its connections to downstream circuit neurons will shed light on the circuits and mechanisms underlying goal-oriented behaviors. They may also reveal whether these mechanisms differ between the larval and adult-fly stages.

It remains unclear whether the number of synapses with certain OSN, PN, and LN partners points to Keystone-LN’s function (Fig. 2). The attractive odorant, 4-hexen-3-one, and the aversive odorant, menthol, used in this study are sensed by OSNs expressing Or42a and Or49a, respectively^22,24,38,39^. According to the larval olfactory circuit connectivity map, Keystone-LN makes at least 4 synaptic connections with OSN::42a (highest among OSNs) and 10 synaptic connections with uPN42a (second highest among PNs) (Fig. 2E,F)^7^. It is, therefore, not surprising that Keystone-LN shows a physiological response to 4-hexen-3-one (Fig. 3B–D) and influences larval behavior responses to the odor (Fig. 4I). In addition, we noted that Keystone-LN manipulations altered larval attraction toward low but not high attractant concentrations. In an active neuronal network, low-frequency stimuli (such as fewer odor molecules in the environment) may induce small but coherent changes in firing rates and timing of neuronal populations that can be magnified by dynamic network activity^47^. Keystone-LN may be activated with higher fidelity by pulses of lower frequencies because of lower action potential generation thresholds than second-order projection neurons (PNs). Keystone-LN could more effectively inhibit PN output in low odor situations, thus slowing down behavior and initiating head sweeps. Conversely, when odor concentration is high, any GABAergic inhibition by Keystone-LN could fail to counteract the high excitatory effects of OSNs on PNs.

Why does a reduction in Keystone-LN’s GABAergic function affect larval aversion to menthol (Fig. 4I)? This is especially intriguing since Keystone-LN has no synaptic contacts with the OSN expressing Or49a, which is most sensitive to menthol (Fig. 2E)^7,39^. Moreover, Keystone-LN barely showed any physiological response to menthol (Fig. 3D). It is possible that Keystone-LN indirectly influences OSN::49a via broad-LNs or picky-LNs in the circuit. We also cannot rule out the possibility of top-down modulation of Keystone-LN’s function in response to aversive odors. These possibilities might suggest a separate computational algorithm to govern orientation behaviors in response to an aversive odor like menthol.

Despite many attempts to study how an animal’s satiety state modulates the function of smell neurons in the mammalian olfactory bulb and the insect antennal lobe^11,13,48–59^, the mechanism by which satiety modulates the function of local neurons in this processing center remains poorly understood. Here we show for the first time that insulin signaling likely mediates the satiety-dependent changes in Keystone-LN, an inhibitory local neuron in the larval antennal lobe (Fig. 5D–H). Insulin is one of the main modulatory hormones that mediate satiety-dependent changes in the sensitivity and attraction to food odors^11,13,60–63^. In the adult fly antennal lobe, insulin signaling adjusts the sensitivity of first-order OSNs via altering presynaptic facilitation. For example, during the animal’s starved state, reduced insulin signaling frees short Neuropeptide-F signaling in first-order olfactory sensory neurons, which leads to increased synaptic facilitation and enhanced attraction to appetitive odors^11–13^. In the vertebrate olfactory bulb, insulin influences the firing rates of second-order neurons by targeting the voltage-gated potassium channel, Kv1.3^48,50–52^. Future studies will uncover precisely how insulin alters Keystone-LN’s function. For example, could insulin signaling impact the inhibitory role of Keystone-LNs by influencing the production of GABA neurotransmitters? Our preliminary quantification of Bruchpilot puncta in Keystone-LNs suggests that insulin signaling might influence the structural integrity and synaptic zone function in Keystone-LNs^40^. It would also be interesting to determine why it is necessary to modulate a circuit’s inhibitory output to implement changes in a goal-directed movement and whether the insulin signaling mechanisms in an inhibitory local neuron are distinct from those in other neurons.

We noted that starved wild-type *Drosophila* larvae and larvae with fewer insulin receptors in Keystone-LN (mimicking a starved state) have smaller magnitude head-sweeps (Fig. 5C,H). Smaller head sweeps could result in starved larvae traveling in straighter tracks (Fig. 5G), allowing them to move farther in less time to search for food. Many crawling insect species have a higher dispersal rate during their starved state. These higher dispersal rates are often accompanied by significantly fewer head wavings^2–5^. For example, starved fifth instar larvae of the cabbage butterfly (*Pieris rapae*) show increased locomotory rate, decreased head-waving, and straighter movements^4,5^. Starved fifth-instars of the Asian mantid (*Paratenodera angustipennis*) switch to active searching^4^. Starved nymphal stinkbugs (*Podisus nigrispinus*) preying on tomato leafminer larvae disperse more and quicker than their satiated counterparts^2^. Starved subjects of the stinkbug (*Bagrada hilaris*) walk farther distances, increasing with longer periods of starvation and moving with more directionality^3^. Interestingly, starved insects appear more willing to expend energy to cover larger distances and search in a pattern with fewer turns. Similar starvation-induced increases in dispersion have been observed in bacteria, amoeboid cells, *Drosophila* larvae, and other crawling and flying insects^1,4,24,64,65^. While a systematic search is most efficient when there is knowledge of food in the general vicinity, traveling further distances in a random direction might be a more efficient search strategy when food is sparse^65^. Thus, it might be more beneficial to the animal if some components of the search behavior (e.g., head sweeps) are adaptive to the situation of the searcher, such as its feeding status. Could hunger activate/deactivate specific pathways (e.g., insulin signaling in Keystone-LNs) that reduce the magnitude of head sweeps, thus allowing the larvae to travel in straighter tracks and disperse more? This adaptation could enable the larvae to adopt an efficient food search strategy, ‘win-stay/lose-shift’, i.e., stay in the presence of food and start moving when starving^66^.

While the satiety-dependent modulation of first-order sensory neurons and second-order neurons in the insect antennal lobe and the mammalian olfactory bulb has been documented, the modulation of local neurons is not well understood. Our study provides, for the first time to our knowledge, evidence to suggest that an animal’s satiety state modulates the function of an olfactory local neuron. Here, we have shown that Keystone-LN, an inhibitory local neuron in the *Drosophila* larval antennal lobe, encodes information about satiety states to adjust head-sweep behavior. Adjusting the magnitude of head sweeps is likely a prelude to decision-making during odor-guided navigation.

Even the relatively simple olfactory circuit of the *Drosophila* larva employs complicated neural algorithms that link circuit activity to behavior toward or away from odors. Furthermore, state-dependent modulation of circuit mechanisms adds further complexity to these algorithms. Understanding this complexity is critical to learning how internal states use the flexibility established in circuits to shape behavioral decisions. In this context, our study on the modulation of Keystone-LN represents a significant step toward bridging the gap between circuit mechanisms and behavior.

## Materials and methods

### Drosophila stocks

Canton-S (CS) was used as the wild-type line in behavioral experiments. UAS-IVS-CsChrimson (Flybase#55135) virgin females were crossed with Keystone-Gal4 (Flybase#49232) males for optogenetic experiments. For immunocytochemistry and behavior experiments, *Keystone-Gal4* virgin females were crossed with males of *UAS-InR-RNAi* (VDRC#992). For two-choice assays, *Keystone-Gal4* virgin females were crossed with *UAS-GAD-RNAi* (Flybase#51794)*.* The *Keystone-Gal4* line, uncrossed, was used as the parent control line. For calcium imaging experiments, *UAS-GCaMP6m; Orco::RFP*^14^ virgins were crossed with *Keystone-Gal4* male flies. Flies were reared at 25 °C and 60% humidity on standard cornmeal-dextrose agar food (Genesee Scientific, #66-112).

### Odorants and other reagents

Test odorants were obtained at the highest purity available (≥ 98% purity; Sigma-Aldrich). Odors were diluted in paraffin oil (Sigma-Aldrich, #76235). Larval crawling surface for behavior experiments was prepared using high-purity Agarose (Genesee Scientific #20-102GP). Odor gradients in the behavior arena were generated by adding odor to 6 mm filter discs (GE Healthcare #2017-006) placed in the arena.

### Behavior assays

#### Preparation of larvae

For all behavior experiments, third-instar larvae (~ 96 h AEL) were extracted from food using a high-density (15%) sucrose (Acros Organics #177140050)^24,36,67^. Larvae floating to the sucrose solution’s surface were then separated and washed four times with distilled water. Washed larvae were allowed to rest for 5 min before subjecting them to behavior assays.

The starvation protocol, when needed, was carried out as described in Slankster et al.^11^. Briefly, washed larvae were allowed to roam freely for 2 h at RT in a 6 cm petri-dish (Falcon Scientific #351007). The petri-dish contained either 350 µL dH_2_O (starved condition) or 350 µL of 0.2 M sucrose (non-starved condition) added to a piece of Kim wipe.

#### Larval two-choice assays

Larval two-choice assays were carried out as previously described^36,37^. Approximately 50 larvae were allowed 5 min in the dark to choose between test odorant or control diluent (paraffin oil) on opposite ends of a 9 cm petridish. After 5 min, larvae on each half of the plate were counted. A Response Index (RI) was calculated using the following formula: *RI* = (*O* − *C*)/(*O* + *C*), where ‘O’ is the number of larvae in the odor half of the plate, and ‘C’ is the number of larvae in the control half of plate.

#### Optogenetics and tracking assays

Optogenetics and tracking assays were carried out as previously described^1,21,24,67^. A video description of the optogenetics assay to stimulate larval neurons and simultaneously record larval behavior was previously published by our lab^21^. *CsChrimson* expressing larval neurons were activated by shining red light (630 nm, 1.3 W/m^2^ intensity, Environmental lights Inc. San Diego, CA, USA) on the behavior arena. The movement of each larva before, during, and after light stimulation was recorded.

#### Data analyses

Analyses of larval trajectories were based on previously published methods^1,24^. Tracks of larvae crossing paths with another were eliminated to control for larva-larva interactions from confounding behavior data. Larvae that exited the main field of view were also excluded from the analysis. Larval trajectories were reconstructed using custom routines written in MATLAB. Trajectories were segmented into a series of ‘runs’ and ‘stops.’ Runs were defined as continuous periods of forward movement. A stop separated successive runs and was flagged when the speed dropped beneath a threshold value (unique to each larva) for more than two frames. Stops were further examined to differentiate between a ‘stop’ and a ‘head sweep.’ A head sweep was flagged if a larva was stopped and the body bend angle was greater than 20°^1^. ‘Normalized crawling speed’ was calculated as the speed of a larva in a given frame(s) (regardless of stops or runs) divided by the average larval speed. The normalized crawling speed was analyzed per frame or averaged over the number of frames in a 6 s span. ‘Probability of head sweep’ was calculated iteratively for each second of the recording as the number of larvae initiating a head sweep divided by the total number of larvae analyzed in the recording. ‘Magnitude of head sweeps’ was calculated in the difference of body bend angle at flexion (highest body bend angle) and extension (lowest body bend angle) for the head-sweep event. ‘Curve rating’ was defined as the ratio of the total length of a trajectory to its actual displacement (a curve rating of 1 indicates a perfectly linear trajectory).

### Calcium imaging

#### Preparation of larvae

Third-instar larvae (~ 96 h AEL) were extracted from food, washed, and subjected to the starvation protocol described above. After this, larvae were washed in Baines’ saline^68^ and dissected in clean Baines’ saline at RT. (Baines’ saline recipe: 135 mM NaCl, 5 mM KCl, 2 mM MgCl_2_, 0.5 mM CaCl_2_, 5 mM *N*-Tris[hydroxymethyl]methyl-2-aminoethanesulfonic acid (TES), and 36 mM sucrose). The posterior two-thirds of larvae were removed from their anterior part, resulting in dissected larvae containing intact olfactory sensory organs and brains. Fat tissues around the brains were carefully removed to reveal the brain lobes. Then the head of the larva was mounted on a poly lysine-coated coverslip dorsal side down with the brain lobes adhered on the coverslip. The larval preparation was set in a drop of 3% agarose with Baines’ saline. Then, the agarose covering the most frontal part of the larva was removed to expose the dorsal organs to air.

#### Airborne odor stimulus delivery

A stimulator (Syntech Stimulus Controller CS55 V2) was used to couple the odor stimulus delivery with imaging. Tubing was set up as seen in Fig. 3A. A constant flow rate at the tube exit of ~ 1.8 L/min is maintained. However, the final flow rate reaching the larval preparation would be lower. Ambient air is filtered through an activated charcoal filter at the air inlet of the stimulator and humidified via bubbling through a gas washing bottle containing dH_2_0 at the outlet. Odor syringes were prepared by inserting a piece of Sugi Sponge Strip with 20 μL of test odorants in 2 mL syringes. They were connected to the pulse flow tubing via an 18G needle going through the syringe plunger (Fig. 3A). The stimulus duration was one second.

#### Imaging

The GCaMP fluorescence signal from Keystone-LN’s neurites in the antennal lobe was imaged on a spinning disk confocal (Yokogawa CSU10 Scanhead and Nikon Eclipse FN1 Microscope Stand) with a CCD camera (Photometrics Evolve 512 EMCCD), a 60 × objective (Nikon Plan Apo VC 60 ×/1.20 WI), and a piezo (MCL Nano-F450). Stacks of images were acquired at a rate of 1 volume per second, each stack consisting of ~ 15 slices with 2 μm intervals. Odors were applied in a random order, except for ethyl acetate, which was applied first to confirm the responsiveness of the larva. The position of the antennal lobe, and thus of the Keystone-LN neurites, was determined by the expression of Orco::RFP labeling. Data were collected from five to six larvae for each condition.

#### Image processing

Fluorescence traces were extracted and processed using a combination of Fiji and Igor scripts. Maximum projections of the volumetric stacks were obtained, movement corrected, and bleach corrected. Fluorescence traces were extracted from manually drawn circular ROIs with the guidance of autocorrelated pixels.

Data analysis of the fluorescence traces was completed in MATLAB.

$$\Delta {\text{F}}/{\text{F}}\;{\text{was}}\;{\text{calculated}}\;{\text{as}}\;\frac{F(t) - F0}{{F0}}$$

*F*(*t*) is the fluorescence at time point *t,* and *F0* is the average baseline fluorescence of 4 frames before the stimulus response. Response amplitudes in ΔF/F were then calculated as $average\; of\;response\; frames - average \;of\; baseline \;frames$. Finally, response amplitudes for test odors were solvent corrected by subtracting paraffin oil (solvent) response amplitude from the test odor response amplitudes.

### Immunocytochemistry

Third-instar larval dissection and antibody staining methods were adapted from^69,70^. GFP was stained using a (1:125) dilution of chicken anti-GFP polyclonal antibody (Invitrogen#PA1-9533). A (1:125) dilution of goat anti-chicken IgY coupled with Alexa Fluor 488 (ThermoFisher#A-11039) was used as the secondary antibody. VGAT was stained using a (1:125) mouse anti-VGAT (Synaptic systems#131011). A (1:125) goat anti-mouse coupled with Alexa Fluor 555 was used as the secondary antibody (Invitrogen#A32727). Insulin receptor was stained using a (1:65) dilution of rabbit anti-InR polyclonal antibody (Cloud-Clone Corporation#PAA895Hu02). A (1:65) dilution of goat anti-rabbit IgG coupled with Alexa Fluor Plus 647 (ThermoFisher#A32733) was used as the secondary antibody. Bruchpilot was stained using a (1:200) mouse anti-BrP (DSHB: Ab 2314866). A (1:500) dilution of Donkey anti-mouse IgG coupled with Cy5 (Jackson Laboratories. RRID: AB_2340820) was used as the secondary antibody. Samples were imaged with a Leica TCS SP8 Confocal Microscope with a 40 × objective. All genotypes were imaged under identical laser power and scan settings.

#### Image analysis

To measure fluorescence intensities, raw images were optimized to select the region of interest (ROIs) and exclude background noise. Fluorescence intensity was measured as the mean intensity of each ROI in raw images for GFP and InR staining using NIH ImageJ (version 1.8.0, Bethesda, MD). The ‘mean integrated intensity’ of GFP (green channel) and InR (red channel) was analyzed for the region of interest (ROIs) by subtracting the background intensity. Bruchpilot puncta within a predefined circular area (1.5 µm^2^) in Keystone-LN terminals were quantified using ImageJ’s ‘analyze particle’ tool.

### Statistical analysis

Statistical analyses were performed using Statistica (StatSoft; RRID: SCR_014213) and R (RRID: SCR_001905). Figure 1: The probability of head sweep was calculated as the number of larvae initiating a head sweep in a given second of recording divided by the total number of larvae analyzed. Randomization analysis was performed as described previously. p-value was adjusted for multiple comparisons using the BH method and calculated using R. The Z-statistic between ‘Lights ON’ and ‘Lights OFF’ was calculated using the Mann–Whitney *U* test in Matlab (RRID: SCR 001622). A randomization test was performed using 1,000,000 repetitions. Figure 3: For the Calcium-imaging experiments, the Mann–Whitney *U* test was used to test whether there was a significant difference between response amplitudes towards odors in fed and starved states. Significance values were set at 0.05. Figure 4: The Shapiro–Wilk test was used to test the assumptions of normality for the probability of head sweep and magnitude of head sweep data. Probability data did not conform to a normal distribution, so a non-parametric analysis equivalent to a two-way ANOVA was used to compare feeding and light conditions (Rfit package in R). Similarly, the magnitude of head sweep data also did not conform to a normal distribution. So, the non-parametric one-way ANOVA equivalent Kruskal–Wallis test was used to analyze differences between fed and starved larvae. Dunn’s test was utilized for posthoc comparisons, and p-values were corrected using the Bonferroni methods (FSA package in R). The normalized crawling speed did not follow a normal distribution. So, a non-parametric analysis equivalent to a mixed-model ANOVA was used (nparLD package in R^71^). Post-hoc analysis was performed with a Wilcoxon sign-rank test between each time increment within each condition. For multiple comparisons, the BH method for controlling false discovery rates was calculated using R^72^. For the 2-choice behavior assay, the normality of the response index was tested with the Shapiro–Wilk test. All samples showed normal distribution, and Levene’s test showed homogeneity of variances. So, a factorial ANOVA with Tukey posthoc analysis was used. Figure 5 and Suppl. Fig. 1: For immunocytochemistry experiments, values comparing the InR and GFP intensity for starved and non-starved conditions for each genotype were calculated using the Student’s *t* test (two-tailed) and plotted using GraphPad Prism (version 9.3.0).

## Supplementary Information

Supplementary Figure 1.

## Data Availability

Key resources used in this study are listed in the methods section. Further information and requests for resources and reagents, raw data, and software codes generated within the present study should be directed to and will be fulfilled by the Lead Contact, Dennis Mathew (dennsimathew@unr.edu).
