# Supplementary material for: Internal state affects local neuron function in an early sensory processing center to shape olfactory behavior in Drosophila larvae
Source: Sci Rep. 2022 Sep 21;12:15767. doi: 10.1038/s41598-022-20147-1 (PMC9492728; doi:10.1038/s41598-022-20147-1)

SUPPLEMENTARY MATERIAL

Internal state affects local neuron function in an early sensory processing center to shape olfactory behavior in *Drosophila* larvae

Seth R. Odell[1¶], David Clark[1¶], Nicholas Zito[1], Roshni Jain[2], Hui Gong[3], Kendall Warnock[4], Ricardo Carrion-Lopez[5], Coral Maixner[5], Lucia Prieto-Godino[3], and Dennis Mathew[1,2,4,5 *]

[1]Integrative Neuroscience Program, University of Nevada, Reno, NV 89557; USA
[2]Molecular Biosciences Program, University of Nevada, Reno, NV 89557; USA
[3]The Francis Crick Institute, London NW1 1AT; UK
[4]Department of Biology, University of Nevada, Reno, NV 89557; USA
[5]NSF-REU (BioSoRo) Program, University of Nevada, Reno, NV 89557; USA
¶These authors contributed equally to this work

***Correspondence**
Corresponding author: Dr. Dennis Mathew
Email: dennismathew@unr.edu
Address: 1664 N. Virginia St., MS: 0314, University of Nevada, Reno, NV 89557; USA

Supplementary Figure 1: Insulin signaling affects the number of Bruchpilot puncta in Keystone-LN terminals.

(**A**) Figure depicting the front end of a third-instar *Drosophila* larva. Keystone-LN is highlighted in green. The rectangular inset marks the region of interest during confocal imaging. α-GFP antibody pinpoints Keystone-LN (left panel). α-BrP antibody labels Bruchpilot (middle panel). The right panel shows the merged image. The top panels are images taken from a parental control genotype. The bottom panels are images of a test genotype expressing InR-RNAi in Keystone-LN. (**B**) The number of BrP puncta in Keystone-LN terminals of control and test genotypes were quantified in a predefined area (1.5 μm^2) (Mean ± SEM). Student's t-test (two-tailed), $p<0.05$.

Supplementary Figure 1

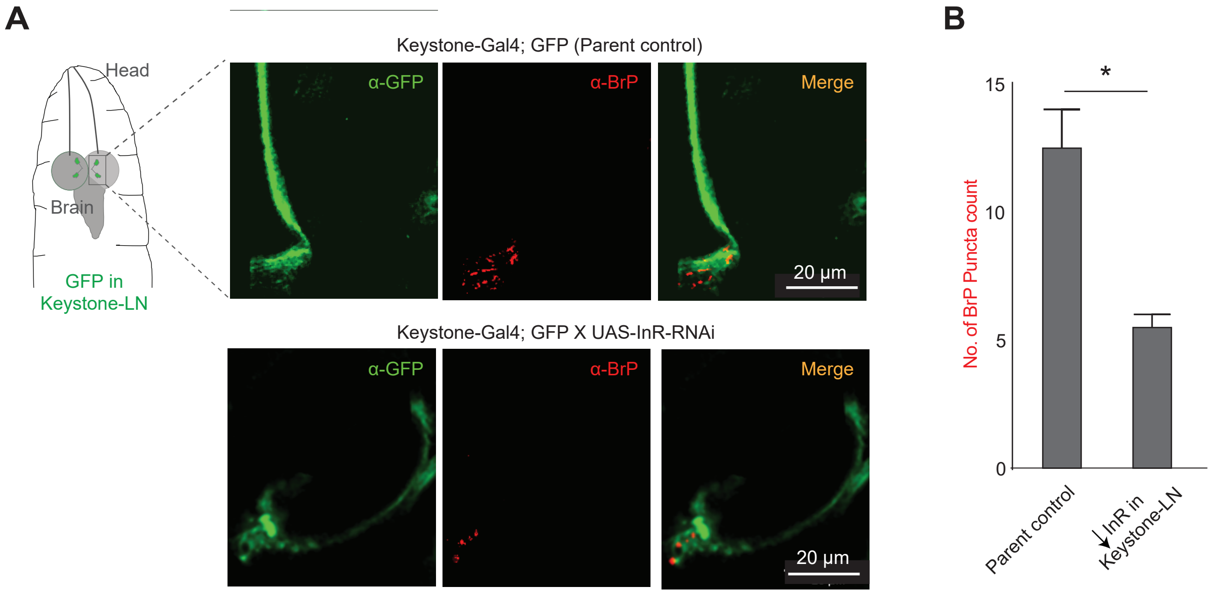

Supplement: Supplementary file 1 — Supplementary Figure 1. [file 41598_2022_20147_MOESM1_ESM.pdf]
